# Delayed gastric emptying after pancreatoduodenectomy: an analysis of risk factors

**DOI:** 10.1007/s13304-024-01795-6

**Published:** 2024-04-10

**Authors:** Juan Carlos Sabogal, Danny Conde Monroy, Carlos Eduardo Rey Chaves, Daniela Ayala, Juliana González

**Affiliations:** 1 Hepatobiliary and Pancreatic Surgery Department, Hospital Universitario Mayor, Méderi, Bogotá, Colombia; 2 School of Medicine, Universidad del Rosario, Bogotá, Colombia; 3 Estudiante de Posgrado Cirugía General, Facultad de Medicina, Pontificia Universidad Javeriana, Carrera 6A #51A-48, 111711 Bogotá D.C, Colombia

**Keywords:** Delayed gastric emptying, Pancreatoduodenectomy, Morbidity, Pancreatic fistula

## Abstract

**Background:**

Delayed gastric emptying (DGE) is a frequent complication after pancreatoduodenectomy. Preoperative factors are limited and controversial. This study aims to identify associated factors related to this complication in the Colombian population.

**Methods:**

A retrospective review of a prospectively collected database was conducted**.** All patients over 18 years of age who underwent pancreaticoduodenectomy were included. Associations with DGE syndrome were evaluated with logistic regression analysis, Odds ratio, and b-coefficient were provided when appropriate.

**Results:**

205 patients were included. Male patients constituted 54.15% (*n* = 111). 53 patients (25.85%) were diagnosed with DGE syndrome. Smoking habit (OR 17.58 *p* 0.00 95% CI 7.62–40.51), hydromorphone use > 0.6 mg/daily (OR 11.04 *p* 0.03 95% CI 1.26–96.66), bilirubin levels > 6 mg/dL (OR 2.51 *p* 0.02 95% CI 1.12–5.61), and pancreatic fistula type B (OR 2.72 *p* 0.02 CI 1.74–10.00).

**Discussion:**

Smoking history, opioid use (hydromorphone > 0.6 mg/Daily), type B pancreatic fistula, and bilirubin levels > 6 mg/dL should be considered as risk factors for DGE.

## Introduction

Pancreatoduodenectomy (PD) remains the milestone treatment in pancreatic cancer [1, 2]. The perioperative mortality of PD in specialized centers is around 0–5%. However, the morbidity remains around 30–50% [3, 4]. Delayed gastric emptying (DGE) is recognized as one of the most frequent complications, presenting between 15 and 40% of the cases [1, 4]. DGE was first described by Warshaw and Torchiana, but no standardized definition was defined until 2007 [5, 6]. The international study group on pancreatic surgery (ISGPS) classified the condition into mild, moderate, and severe based on the nasogastric tube necessity and time to tolerate food intake [7, 8]. In patients taken to PD, it is the leading cause of increased morbidity, prolonged hospital length of stay, and readmission, with an incidence of around 25–75% [9, 10].

Although the pathophysiological mechanisms involved in developing DGE are not yet elucidated, hypotheses related to preoperative, intraoperative, and postoperative factors have been described [11, 12]. Some of the attributed mechanisms include the loss of the pylorus, damage to vagal fibers, local ischemia, or lack of motilin secreted in the duodenum [10–12]. Therefore, several variations in techniques have been attempted to improve the unwavering incidence of DGE. Among the interventions with the promising results is the kind of enteric reconstruction (ante colic instead of retro colic) [13, 14], the preservation or resection of the pylorus (classic Whipple vs. Pylorus preserving PD) or the pancreatic enteric anastomosis (pancreatic-jejunum vs. pancreatic-gastric) [14, 15]. However, the results are mixed, and many of the studies are small cohorts with retrospective analyses that lack consistency in the definition of DGE [15, 17].

Despite the large amount of data available on DGE, information about the clinical and preoperative factors associated with this complication and data from Latin America is scarce [17, 18]. Reliable preoperative predictors are lacking to improve patient outcomes and to create preventative management of this serious condition [18]. This study aims to evaluate the DGE presentation rate, clinical features, and the associated factors with DGE in patients taken to classic Whipple surgery between 2014 and 2022 in a center for hepatobiliary and pancreatic surgery in Colombia.

## Methods

### Study population

A retrospective review of a prospectively collected database was conducted with the Institutional (Hospital Universitario Mayor Méderi) Review Board’s approval and following Health Insurance Portability and Accountability Act (HIPAA) guidelines. All patients over 18 years of age who underwent pancreaticoduodenectomy for all causes were included. Patients with missing data were excluded. Perioperative variables included demographics, associated comorbidities, previous mal-nutrition state, pyloric obstruction, parenteral nutrition, and previous bile duct drainage. Some operative variables: operative time and blood loss. And postoperative variables: Surgical site infection, postoperative pancreatic fistula, and opioid use. Technical characteristics of the surgery were ruled out as variables because the surgical technique is standardized for all cases. Ethical compliance with the Helsinki Declaration, current legislation on research Res. 008430-1993 and Res. 2378-2008 (Colombia), and the International Committee of Medical Journal Editors (ICMJE) were ensured under our Ethics and Research Institutional Committee (IRB) approval.

### Sample size

Sample size calculation was performed following Freeman’s model for unconditional logistic regression. Considering the delayed gastric emptying rate of 27% of our population and with an estimated dropout of 10%, the total sample size calculation was 205 patients.

### Data management: statistical analysis

Descriptive statistics were reported in terms of variable nature. Normality was evaluated using the Shapiro–Wilk test. Qualitative analysis was performed in terms of frequencies and percentages, while quantitative analysis was done in terms of mean and standard deviations of normally distributed data and medians and interquartile ranges (IQRs) for non-normally distributed data. Based on the coefficient of variation (CV), the homogeneity of the quantitative variables was evaluated; if CV < 10% was assumed to be homogeneous, 10–20% was moderately homogeneous, and > 20% was heterogeneous. The association between demographic, clinical, and preoperative variables was evaluated using the chi-squared Pearson test, likelihood ratio, or Fisher exact test (Expected values less than 0.2), and association strength was assessed with relative risk with a 95% confidence interval. For quantitative variables, the homogeneity of the variables was evaluated using the Bartlett–Box test, and in cases of normal distribution, Student t test for two independent groups was performed. Otherwise, a nonparametric Mann–Whitney test was used. The overall group associations with delayed gastric emptying syndrome were evaluated with logistic regression analysis, odds ratio, and b-Coefficient were provided when appropriate. Statistically significant value was accepted if *P* value < 0.05. Statistical analysis was performed in Stata version 18.0.

### Surgical technique

As a standardized classic technique, pancreatoduodenectomy is performed clockwise and divided into three parts: dissection, resection, and reconstruction. for the dissection part, a Kocher maneuver is made until visualization of the left renal vein, hepatoduodenal dissection, and lymph node retrieval, the hepatic artery lymph node is resected and sent to pathology, dissection, and repair of the gastroduodenal artery, gastrocolic aperture, and pancreatic neck dissection until a tunnel is created between the pancreas and superior mesenteric vein. The resection phase is made in this order, cholecystectomy, bile duct section over cystic duct, gastroduodenal artery ligation (always checking for no replacement of the hepatic artery), gastric section at 2 cm above pylorus, pancreatic resection using cautery and hemostatic stitches, and finally jejunal resection using line stapler at 10–15 cm below Treitz Ligament. The resection is completed by transection of the fixation of the uncinate process to the mesenteric vasculature. The final phase is the reconstruction. A transmesocolic ascent of distal jejunum is performed for a pancreato-jejunostomy and hepatico-jejunostomy. The first one is performed in a duct-mucosa technique in 2 layers with a probe left inside the anastomosis. Hepatico-jejunostomy is performed by a modified Blumgart technique at 15–20 cm distal to the PJ anastomosis. We use polydioxanone 4.0 or 5.0 in both anastomoses. Finally, an ante colic gastro-jejunostomy is made in two planes by hand at 60 cm of ascending asa.

### Pancreatic fistula risk assessment

A pancreatic fistula score measures the risk of pancreatic fistula and decides to leave an active drain. We use the Fistula Risk Score (FRS) to calculate the risk of pancreatic fistula, which is available in an app. This scale was defined by Callery et al. [18], based on a homogeneous series of patients, it uses four parameters that include consistency of the pancreatic gland, histopathology of the resected pancreas, the diameter of the Wirsung’s duct and intraoperative bleeding volume. According to the multivariate analysis performed, only soft pancreatic parenchyma, the presence of ampullary, duodenal, cystic, or islet cell pathology, a pancreatic duct diameter equal to or less than 3 mm, and intraoperative bleeding greater than 1000 were significant (3-times increased risk of developing CR-POPF).

The score can oscillate between 0 and 10 points and is stratified into very low risk (0 points), low risk (1–2 points), intermediate risk (3–6 points), and high risk (7–10 points) [18].

### DGE diagnosis and assessment

DGE was diagnosed using the ISGPS classification [7]. Grade A: the requirement of a nasogastric tube (NGT) for 4–7 days, or reinsertion after POD 3, unable to tolerate solid oral intake by POD 7, grade B: the requirement of a nasogastric tube (NGT) for 8–14 days, or reinsertion after POD 7, unable to tolerate solid oral intake by POD 14. Grade C: as the requirement of a nasogastric tube (NGT) for > 14 days, or reinsertion after POD 14, unable to tolerate solid oral intake by POD 21. For grades B and C, enteral or parenteral nutrition was used as part of the treatment [7, 13, 18].

## Results

### Cohort

A total of 205 patients underwent pancreatoduodenectomy between 2014 and 2022, matching the inclusion criteria and being included in our study. A total of 53 patients (25.85%) were diagnosed with delayed gastric emptying syndrome independent of the grade. Grade A DGE was present in 16.59% (*n* = 34) of cases, grade B in 4.88% (*n* = 10), and 3 patients with DGE grade C.

### Demographics and clinical characteristics

Most of the patients were male 54.15% (*n* = 111). The median age was 66 years old (IQR 58; 73). The mean BMI was 23.32 (IQR 14.9–35.21) Kg/m^2^. History of hypertension was presented in 39.02% (*n* = 80), T2DM in 21.95% (*n* = 45) of the patients, and a history of smoking in 37.56% (*n* = 77) of the population. Coronary illness was documented in 7.80% (*n* = 16) of the patients. (Table 1).

**Table 1:** Patient’s characteristics

Variable	Result
Age median (IQR)	66 (58; 73)
Body mass index median (IQR)	22.80 (20.89; 25.5)
Gender % (*n*)	
Male	54.15 (111)
Female	45.85 (94)
Smoking history % (*n*)	37.56 (77)
T2DM % (*n*)	21.95 (45)
Arterial Hypertension % (*n*)	39.02 (80)
Coronary Arterial disease % (*n*)	7.80 (16)
Preoperative bile duct drainage % (*n*)	37.56 (77)
Preoperative parenteral nutrition % (*n*)	6.83 (14)
Total bilirubin median (IQR)	4.64 (1.04;10.04)
Operative time median (IQR)	290 (240;330)
Use of opioids (hydromorphone) > 0.6 mg % (*n*)	94.15 (193)
Tumor size > 2 cm % (*n*)	78.54 (161)
Previous malnutrition diagnosis % (*n*)	46.83 (96)
Preoperative pyloric syndrome % (*n*)	4.88 (10)
Pancreatic fistula % (*n*)	28.78 (59)
Biochemical leak % (*n*)	18.54 (38)
Type B pancreatic fistula % (*n*)	8.29 (17)
Type C pancreatic fistula % (*n*)	1.95 (4)
Intra-abdominal collections % (*n*)	10.24 (21)
Surgical site infection % (*n*)	6.34 (13)
Confirmed malignant diagnosis % (*n*)	84.80 (173)
Delayed gastric emptying	
Overall % (*n*)	25.85 (53)
Type A % (*n*)	16.59 (34)
Type B % (*n*)	4.88 (10)
Type C % (*n*)	1.46 (3)
In-hospital stay mean (SD)	17.76 (12.2)
Re-admission rate % (*n*)	8.78 (18)

In terms of preoperative variables, 46.83% (*n* = 96) of the patients were classified with preoperative malnutrition (defined as loss of greater than 5% of the corporal weight); 4.88% (*n* = 10) of the patients underwent pancreatoduodenectomy in the scenario of pyloric obstruction due to tumor infiltration. Malignancy was confirmed in 84.80% (*n* = 173) of the patients, and in this case, tumor size was classified in most of the population as greater than 2 cm in 78.54% of the population. Median serum bilirubin from the previous surgery was 4.64 mg/dL (IQR 1.04; 10.04), and in 37.56% of the cases, patients required bile duct drainage before surgery. The median operative time was 290 min (IQR 240; 330).

Regarding the postoperative variables, 94.15% (*n* = 193) of the population requires more than 0.6 mg per day of opioids to control postoperative pain. Postoperative outcomes were included as variables; overall postoperative pancreatic fistula was 28.78% (*n* = 59); and in most of the patients was classified as a biochemical leak according to the international classification. 10.24% (*n* = 21) of the patients were diagnosticated with intra-abdominal collections. The surgical site infection rate was 6.03%. The mean hospital length was 17.76 ± 12.2 days, and the readmission rate of any condition was 8.78% (n = 18). Summarized data are displayed in Table 1.

Differential analysis by population regarding the presence of delayed gastric emptying was performed; both groups were similar in demographic, clinical, and operative characteristics. With slight differences regarding the presence of pancreatic fistula. Also, in-hospital length of stay was notably higher in patients with DGE (23.03 vs. 15.9 days). Summarized data are displayed in Table 2.

**Table 2:** Comparative analysis regarding DGE

Variable	No DGE (*N* = 152)	DGE (*N* = 53)
Age median (IQR)	65 (58–72)	67 (57–74)
Body mass index median (IQR)	22.91 (20.6–25.6)	22.6 (21.23–24.65)
Gender % (*n*)		
Male	53.28 (81)	56.60 (30)
Female	46.72 (71)	43.40 (23)
Smoking history % (*n*)	22.36 (34)	81.13 (43)
T2DM % (*n*)	22.36 (34)	20.75 (11)
Arterial Hypertension % (*n*)	42.10 (64)	30.28 (16)
Coronary arterial disease % (*n*)	7.23 (11)	9.43(5)
Preoperative bile duct drainage % (*n*)	40.13 (61)	30.18 (16)
Preoperative parental nutrition % (*n*)	5.92 (9)	9.43 (5)
Total bilirubin median (IQR)	3.50 (0.8–9.6)	6.7 (2.7–12.2)
Operative time Median IQ	282.5 (240–332)	300 (240–329)
Use of opioids (hydromorphone) > 0.6 mg % (*n*)	92.76 (141)	98.11 (52)
Tumor size > 2 cm % (*n*)	80.92 (123)	71.69 (38)
Previous malnutrition diagnosis % (*n*)	47.36 (72)	45.28 (24)
Preoperative pyloric syndrome % (*n*)	2.63 (4)	11.32 (6)
Pancreatic fistula % (*n*)	26.31(40)	35.84 (19)
Biochemical leak % (*n*)	75 (30)	42.10 (8)
Type B pancreatic fistula % (*n*)	20 (8)	47.36 (9)
Type C pancreatic fistula % (*n*)	5 (2)	10.52 (2)
Intra-abdominal collections % (*n*)	8.55 (13)	42.10 (8)
Surgical site infection % (*n*)	5.92 (9)	7.54 (4)
Confirmed malignant diagnosis % (*n*)	83.55 (127)	86.79 (46)
In-hospital stay mean (SD)	15.9 (9.76)	23.03 (16.45)
Re-admission rate % (*n*)	6.57 (10)	15.09 (8)

### Associated factors: statistical analysis

The initial analysis demonstrated that smoking habit was related with statistically significant value with any grade of delayed gastric emptying syndrome (Chi 57.86 *p* = 0.00) and arterial hypertension is associated as well (CHI 2.34 *p* = 0.12). The use of opioid therapy with more than 0.6 mg of hydromorphone daily shows a statistical relationship with DGE (Chi2 2.04 *p* = 0.15). Tumor size is also associated with a statistically significant value (Chi2 1.98 *p* = 0.15). The total bilirubin value was related to DGE (Z − 2.1 *p* = 0.02) and preoperative biliary drainage (Chi 1.65 *p* 0.19). The presence of pancreatic fistula in any grade shows a relationship with DGE (CHI 1.74 *p* = 0.18), and especially Type B pancreatic fistula (Chi 7.09 *p* ≤ 0.00), intra-abdominal collections are related with statistically significant value (Chi2 1.82 *p* = 0.17). Bilirubin levels were evaluated using an ROC Curve, identifying that a total bilirubin level greater than 6 mg/dL shows a positive likelihood ratio of 1.5. A secondary analysis categorizing the variable shows a statistically significant association (Chi2 4.58 *p* = 0.03). All of these variables contribute independently to the presentation of DGE. (Table 2).

In a final multivariate analysis, variables that show statistically significant association with DGE were, Smoking habit (OR 17.58 *p* 0.00 95% CI 7.62–40.51), hydromorphone use greater than 0.6 mg/daily (OR 11.04 *p* = 0.03 95% CI 1.26–96.66), Bilirubin levels > 6 mg/dL (OR 2.51 *p* = 0.02 95% CI 1.12–5.61), and pancreatic fistula type B (OR 2.72 *p* = 0.02 CI 1.74–10.00), see Table 3.

**Table 3:** Initial statistical analysis

Variable	Chi2	Z score	*p* value
Gender	0.17		0.67
Age		(−) 0.47	0.63
Body mass Index		0.20	0.83
Smoking History	57.86		*0.00*
T2DM	0.05		0.80
Arterial hypertension	2.34		*0.12*
Coronary Arterial Disease	0.26		0.60
Operative time		0.02	0.97
Opioids (hydromorphone) > 0.6 mg/Daily	2.04		*0.15*
Tumoral size > 2 cm	1.98		*0.15*
Preoperative malnutrition	0.06		0.79
Pancreatic fistula	1.74		*0.18*
Biochemical leak	0.56		0.45
Type B pancreatic fistula	7.09		*0.00*
Type C pancreatic fistula	1.24		0.26
Intra-abdominal collections	1.82		*0.17*
Surgical site infection	0.17		0.91
Preoperative bile duct drainage	1.65		*0.19*
Total bilirubin		(−) 2.1	*0.02*
Preoperative parenteral nutrition	0.76		0.38
Confirmed malignancy	0.21		0.63

## Discussion

Although it is not a life-threatening complication, DGE has been associated with increased morbidity, prolonged hospitalization, and higher estimated public health costs of up to 10,000 USD in the United States in the presence of other complications [3, 6, 15, 16]. Overall, in this study, DGE was present in 25.86% of pancreatoduodenectomies performed. 16.59% was classified as grade A, 4.88% as type B, and 1.46% as type C DGE (Table 4).

**Table 4:** Final statistical analysis

Variable	OR	*p* value	B Coefficient
Smoking History	***17.58***	***0.00***	***2.83***
Arterial hypertension	1.29	0.56	
Opioids (hydromorphone) > 0.6 mg/Daily	***11.04***	***0.03***	***2.40***
Tumoral size > 2 cm	0.68	0.42	
Pancreatic fistula	1.2	0.62	
Type B pancreatic fistula	***2.75***	***0.01***	***1.00***
Intra-abdominal collections	1.6	0.43	
Preoperative bile duct drainage	0.68	0.38	
Bilirubin > 6 mg/dL	***1.06***	***0.02***	***0.99***

Although the mechanisms are multifactorial, most efforts have traditionally focused on surgical technique modifications. Several studies have compared classic pancreatoduodenectomy (cPD) vs. pylorus-preserving pancreatoduodenectomy (ppPD) as a relevant factor in DGE [13, 19]. Wu et al. [21] documented a meta-analysis where 2451 patients were studied, 1209 with cPD and 1242 with ppPD. They found no significant difference in DGE between the two techniques (95% CI: 0.52–1.17) [20]. Hanna et al. [22] performed a meta-analysis that included 8 studies with a total of 663 patients, comparing SSPPD (subtotal stomach preserving pancreaticoduodenectomy) vs. ppPD (Pylorus preserving pancreatoduodenectomy) reporting a lower incidence of DGE in SSPPD (21%) vs. ppPD (37%) with morbidity rates of 35% vs. 31%, with no significant differences in postoperative complications [21, 22].

The anatomical configurations of reconstruction are also thought to be important for gastric emptying. Antecolic reconstruction seems to be a protective factor against DGE [23]. A meta-analysis performed in the United States included 1067 patients undergoing ppPD, 504 patients underwent ante colic reconstruction (AC), and 563 underwent retro colic reconstruction (RC), revealing a lower incidence of DGE in AC reconstruction of 12.1% and 20.7% in RC reconstruction [22, 24]. Kurahara et al. found that the overall incidence of DGE in the ante colic group was lower than in the RC reconstruction group [24]. However, multiple retrospective studies have not observed significant differences, requiring more studies [25, 26]. In our institution, there is a standardized technique that we can summarize in the following points: use of a duct-mucosa pancreatic enteric anastomosis and trans- stent, ante colic gastric-jejunum reconstruction and the section of the stomach 2 cm above the pylorus (to preserve gastric nerves). Despite being in line with the most accepted surgical techniques, the incidence is still a matter of concern.

Therefore, we believe in the value of identifying preoperative risk assessment. This facilitates counselling, treatment, and adjustment of modifiable risk factors. In this study, the most relevant preoperative factors associated with DGE were smoking habit, opioid daily use greater than 0.6 mg/dL, high bilirubin levels, and the presence of type B pancreatic fistula.

Regarding the relationship between smoking habit and the risk of DGE, the studies are inconclusive and limited [14, 15, 27]. We identify an association between smoking habit and DGE in the analysis (OR 17.58, *p* ≤ 0.00 95%, CI 95% 7.62–40.51). Along the same lines, some authors identified that patients with a smoking history have a higher risk than nonsmokers [6, 28]. However, the outcomes were not clinically significant and had a low statistical correlation because smoking didn’t affect gastric motility by itself. On the other hand, a recent retrospective study in Germany of 274 subjects showed that active smokers have a lower incidence of DGE and present a better tolerance to solid food, acting as a protective factor [15]. Our results align with the hypothesis that its negative effect on the vessel might lead to reduced gastric vascularization and motility [29].

Another relevant fact in this study was the association between analgesic therapy and the development of DGE. Usually, the pre-, peri- and postoperative analgesic management of PD is based on the enhanced recovery after surgery (ERAS, fast-Trak, or Clinical Pathways), which are multimodal strategies that aim to attenuate the loss of and improve the restoration of functional capacity after surgery, emphasizing pain management as a critical parameter in optimal postoperative recovery [30]. Thoracic epidural anesthesia (EDA) remains the gold standard method for major open abdominal surgery (PD) [31]; however, there are instances where patient-controlled intravenous analgesia (PCIA) with or without opioids is used [30, 31].

According to ERAS [30], it has been documented that EDA allows attenuating metabolic and endocrine responses induced by stress; additionally, increased levels of perioperative hypotension have been described, leading to hemodynamic instability, compromising enteric anastomoses, intestinal perfusion, and gastrointestinal function, increasing risk of surgical complications due to the increased fluid demand and the use of vasopressor support, both of which are related to the development of DGE [32]. Prat et al. [33] reported a 15% reduction in postoperative gastrointestinal complications when using EDA vs. PCIA, Wande et al. [34] described an incidence of DGE of 8% in patients with PCIA vs. 9% in patients with EDA, leading to higher use of parenteral nutrition in up to 16% of cases. Despite these postulated benefits, the influence of EDA on clinically relevant outcomes is not clear; most trials to date have not correlated analgesic treatment with surgical complications [30, 33, 34].

Opioids have been the first analgesic management described; however, they have been associated with multiple adverse effects, including postoperative complications such as DGE and postoperative ileus [35, 36]. Numerous studies have shown that the opioid-sparing strategy results in accelerated gastrointestinal recovery with decreased incidence of DGE and shorter hospital stays. In our cohort, epidural analgesia was not used frequently. According to our study, 94.15% of patients (*n* = 193) were dosed with more than 0.6 mg/day of hydromorphone, which was directly related to the development of DGE (OR 11.04, *p* = 0.03; 95% CI 1.26–96.66).

A study conducted in patients who developed DGE without other intraabdominal complications shows that a higher bilirubin level is a risk factor; notwithstanding, in patients with another intraabdominal condition (like a fistula or intraabdominal collection), the bilirubin is not a significant risk factor, but the preoperative biliary drainage behaves like a protective factor, according to different research studies [37, 38, 39], nonetheless, the evidence is inconclusive, and indications for the preoperative drainage are limited. Our study reported an association between the development of DGE with elevated bilirubin levels > 6 mg/dL (IQR 1.04–10.04) and a history of intra-abdominal drainage (Chi 1.65, *p* = 0.19).

Multiple studies associate DGE with pancreatic fistula [11, 40–42]. Patients who presented with POPF have an increased incidence of DGE (31% vs. 13%) and an increased risk of presenting DGE (OR 2.66 CI 1.65–4.28 *p* < 0.001). These data reflect a significant association between POPF and DGE [43, 44]. Our study evidenced that 28.78% (*n* = 59) of the patients presented POPF, 18.54% (*n* = 38) were a biochemical leak, 8.29% (*n* = 17) were type B, and 1.95% (*n* = 4) were type C. According to our results, the postoperative complication with the highest DGE association was type B POPF (OR 2.72 *p* 0.02 CI 1.74–10.00).

The data regarding the Latin American population is scarce. Nevertheless, available evidence shows morbidity rates range between 25 and 60% [20, 45] in most of the series due to the development of postoperative pancreatic fistula (POPF); also, delayed gastric emptying with a range between 12 and 44%; [44–46, 47]. Even though there is no clear relationship with mortality, one study shows an independent association between DGE and mortality (OR 3.25, 2.16–4.88, *p* < 0.001) [44], consequently, all efforts should be focused on its prevention and opportune treatment.

Our study has several limitations that should be mentioned, first a selection bias can occur due to the retrospective nature of the study. The population involved was treated in only an institution by a unique group and with a standardized technique, therefore the influence in other surgical strategies cannot be evaluated.

## Conclusion

In conclusion, DGE is a condition associated with high morbidity. All efforts to prevent this complication should be assessed. According to our data, smoking history, opioid use (hydromorphone > 0.6 mg/Daily), type B pancreatic fistula, and bilirubin levels > 6 mg/dL should be considered risk factors for DGE, and some strategies can be added to reduce them. Nevertheless, further prospective studies with larger sample sizes must validate our results.

## Data Availability

The datasets used and/or analyzed during the current study are available from the corresponding author upon reasonable request.
